# Structure and Properties of Copper Pyrophosphate by First-Principle Calculations

**DOI:** 10.3390/ma15030842

**Published:** 2022-01-22

**Authors:** Anna Majtyka-Piłat, Marcin Wojtyniak, Łukasz Laskowski, Dariusz Chrobak

**Affiliations:** 1 Institute of Materials Engineering, Faculty of Science and Technology, University of Silesia in Katowice, 75 Pułku Piechoty 1A, 41-500 Chorzow, Poland; dariusz.chrobak@us.edu.pl; 2 Institute of Physics—Center for Science and Education, Silesian University of Technology, Krasińskiego 8, 40-019 Katowice, Poland; marcin.wojtyniak@polsl.pl; 3 Institute of Nuclear Physics Polish Academy of Sciences, 31-342 Cracow, Poland; lukasz.laskowski@ifj.edu.pl

**Keywords:** nanocrystals, nanoreactors, DFT, electronic properties, magnetic properties

## Abstract

Investigated the structural, electronic, and magnetic properties of copper pyrophosphate dihydrate (CuPPD) by the first-principle calculations based on the density functional theory (DFT). Simulations were performed with the generalized gradient approximation (GGA) of the exchange-correlation functional (*E_xc_*) supplemented by an on-site Coulomb self-interaction (*U*–Hubbard term). It was confirmed that the GGA method did not provide a satisfactory result in predicting the electronic energy band gap width (*E_g_*) of the CuPPD crystals. Simultaneously, we measured the *E_g_* of CuPPD nanocrystal placed inside mesoporous silica using the ultraviolet–visible spectroscopy (UV–VIS) technique. The proposed Hubbard correction for Cu-3d and O-2p states at *U* = 4.64 eV reproduces the experimental value of *E_g_* = 2.34 eV. The electronic properties presented in this study and the results of UV–VIS investigations likely identify the semiconductor character of CuPPD crystal, which raises the prospect of using it as a component determining functional properties of nanomaterials, including quantum dots.

## 1. Introduction

In recent years, nanocrystalline materials, including quantum dots (QDs), have attracted great interest in various scientific areas, such as nanophotonics, electronics, mechanics, catalysis, and medicine. Their unique properties, essentially broad excitation spectra, narrow emission bandwidth, size-dependent tunable fluorescence, high quantum yield, and high photostability, raise the prospect of using them as a critical component of biosensors, chemical sensors [1,2], light-emitting diodes [3], or ultra-low lasing threshold lasers [4]. Moreover, numerous kinds of nanoparticles (NPs) have been used extensively in medicine as biocidal agents, disinfectants [5], or therapeutic and diagnostic purposes [6]. Although the electric and magnetic properties of many semiconductor QDs have been extensively studied and well recognized, the structure and properties of metal pyrophosphate compounds existing in the nanocrystalline form are less explored and often much more complex.

The pyrophosphate groups, also known as condensed phosphates, play numerous essential roles in the biochemistry of living organisms [7]. Interestingly, phosphonate nucleotide analogs can inhibit SARS-CoV-2 RNA polymerase, making them crucial antiviral agents [8]. Furthermore, in addition to biomedical applications, phosphates, mainly those containing metal, are widely used in many other areas, such as energy science [9], sensors [10], or catalysis [11]. Such molecules can be separated and placed precisely inside porous silica structures [12,13,14]. The materials obtained in such a way can be a starting point for the fabrication of nanocomposites composed of copper pyrophosphate and silver oxide nanocrystals placed inside mesoporous silica of the SBA-15 type, as we showed in our previous works [15].

Theoretical modeling provides an essential insight into the atomic structure and nanoscale phenomena, which has become a significant means for complementing experiments. Quantum mechanical calculations, such as density functional theory (DFT) [16,17], are routinely used to investigate and understand the structural, magnetic, mechanic, optical, and electronic properties of many different materials [18,19,20]. For structure property calculations of our CuPPD crystal, we selected the generalized gradient approximation (GGA) correlation energy functionals (XC) in the form parameterized by Perdew–Burke–Ernzerhof (PBE) [21], which remains the most satisfactory for solids containing 3d transition elements [22]. However, DFT based on GGA does not adequately describe the electronic and magnetic structure of materials containing 3d or 4f states. Therefore, these characteristics are studied based on DFT supplemented by strong Coulomb interaction via Hubbard-like Hamiltonian (U) [23]. The above strategy is already recognized to be capable of minimizing a deficiency of the DFT calculation, which underestimates the semiconductor band gap [23].This article reviews the theoretical investigation of the ground state (i.e., magnetic and electronic properties), as well as the value and character of the band gap of semiconductor-like copper pyrophosphate material. Our spin-polarized DFT calculations reveal the presence of a narrow peak in the spin-up and spin-down channels concentrated around and slightly above the Fermi energy, which constitutes that nanocrystalline copper pyrophosphate exhibits a semiconductor-like character and appears to be a good candidate for QD material. Furthermore, we report the influence of the 3d states of copper metal on the magnetic properties of CuPPD material. The DFT and DFT+U theoretical predictions for the energy and character of the band gap were compared with experimental investigation contributed by UV–VIS measurements. To the best of our knowledge, it is the first time that the suitability of the DFT+U to investigate m-CuPPD nanocrystal and its bulk counterpart was discussed and demonstrated. Therefore, our results propose an efficient and beneficial scheme for promoting new practical knowledge in theoretical simulations of similar compounds of technology relevance. Furthermore, our well-tuned DFT approaches may be applied to many other specific nanocrystalline materials.

The following section describes the experimental and computational methods used in the calculation. The main results are presented and discussed in Section 3. The paper ends with a summary and some conclusions.

## 2. Experimental and Computational Details

The preparation of the nanocrystalline copper pyrophosphate in silica nanoreactors is presented in our article [15]. The DFT studies were carried out using the Quantum Espresso code [24]. The calculations were performed using the generalized gradient approximation (GGA) correlation energy functionals (XC) in the form parameterized by Perdew–Burke–Ernzerhof (PBE) [21]. The XC energy functional was supplemented by strong Coulomb interaction via Hubbard-like Hamiltonian [23]. In the Hubbard model, the effective LDA+U energy functional is written as:

(1) $$E^{\mathrm{LDA}+U}\left[n\right]=E^{\mathrm{LDA}}\left[n\right]+E^{U}n_{i}^{\sigma }-E^{\mathrm{dc}}n_{i}^{\sigma }$$

where E^LDA^ is the standard LDA(GGA) energy functional; E^U^n_i_^σ^ denotes the Hubbard interaction energy of the localized correlated orbitals (typically localized d or f orbitals); E^dc^n_i_^σ^ defines double counting term, which cancels the electron–electron interaction in the localized shell within LDA/GGA; and n_i_^σ^ represents the particle density matrix.

A kinetic energy cutoff of 65 Ry and a charge density cutoff of 325 Ry were assumed for PBE calculations. Similarly, values of 68 Ry and 325 Ry were used in the case of PBE+U studies. Optimized crystal structures were obtained by relaxing atomic positions and cell parameters under the Broyden–Fletcher–Goldfarb–Shanno (BFGS) minimization scheme [25]. A 7 × 7 × 12 Monkhorst–Pack mesh [26] in reciprocal space enabled us to achieve a well-converged total energy of the system as well as its atomic configurations. Electronic properties were calculated using a dense 12 × 12 × 20 Monkhorst–Pack grid. To investigate the magnetic stability, we made calculations for the ferromagnetic (FM) and antiferromagnetic (AFM) configuration. To account for the enhanced Coulomb correlation for Cu-3d and O-2p electrons, we used the DFT+U formalism [23]. The Hubbard term U(2–8 eV) was utilized to improve the interaction between electrons occupying the d and p orbitals of Cu and O atoms, respectively.

The energy gap of copper pyrophosphate was investigated using the ultraviolet–visible spectroscopy (UV–VIS) technique. We have used a microspectrophotometer from CRAIC Technologies (San Dimas, CA 91773, USA) equipped with a standard halogen lamp and Zeiss 15× objective. The sample was cold-pressed into a very thin pellet or a flake. The experiments were performed at room temperature and ambient pressure. To estimate the band gap width, we used the formula proposed by Wood and Tauc [27]:

(2) $$h\nu \times \alpha \sim {\left(h\nu -E_{g}\right)}^{n}$$

where *α* is the absorbance, *h* stands for the Planck constant, *ν* defines the photon’s frequency, *E_g_* denotes the optical band gap energy, and *n* is a constant related to different electronic transitions. The *n* parameter equals 0.5, 2, 1.5, and 3 for direct, indirect, allowed, and forbidden transitions, respectively.

## 3. Results and Discussion

**Figure 1:**
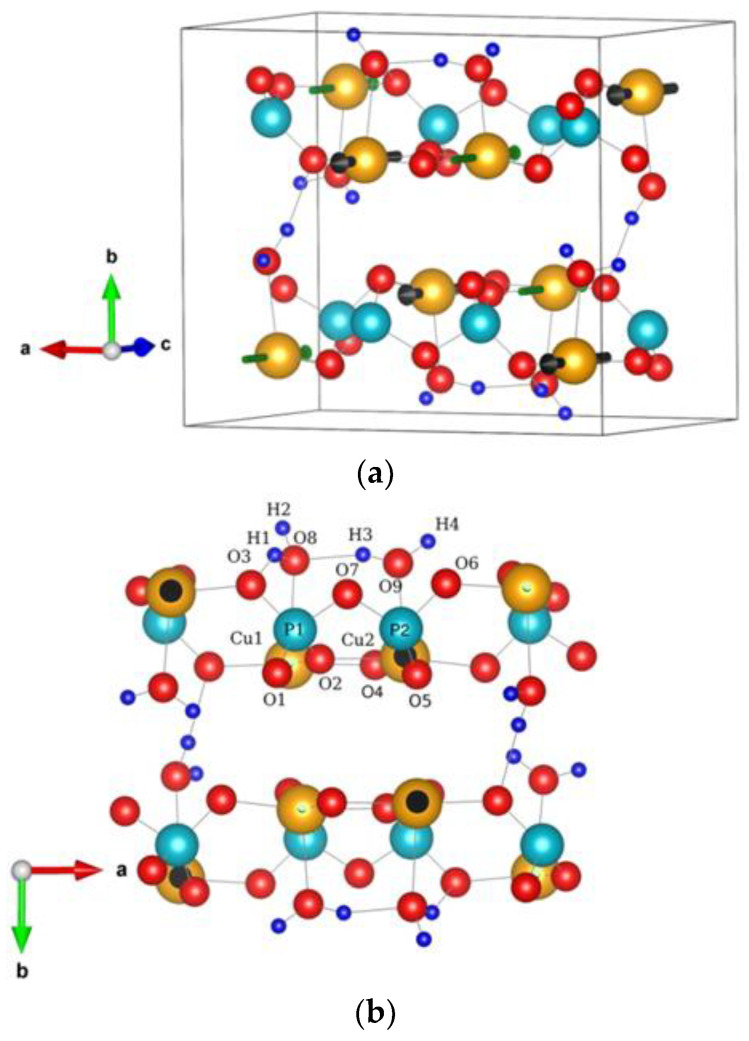
(**a**) Crystal structure of Cu_2_P_2_O_7_(H_2_O)_2_. The orange, blue, red, and navy blue spheres denote copper, phosphorus, oxygen, and hydrogen atoms. Black and green arrows indicate the spin magnetic moment direction of the Cu atoms. (**b**) CuPPD crystal structure viewed from the y-direction showing the details of atomic arrangement.

According to the experimental results provided by Gras et al. [28], the structure of Cu_2_P_2_O_7*_2H_2_O (m-CuPPD) crystallized in a P_21_/n space group includes pyrophosphate ions and two water molecules (Figure 1). Each formula unit is repeated four times per cell.

**Figure 2:**
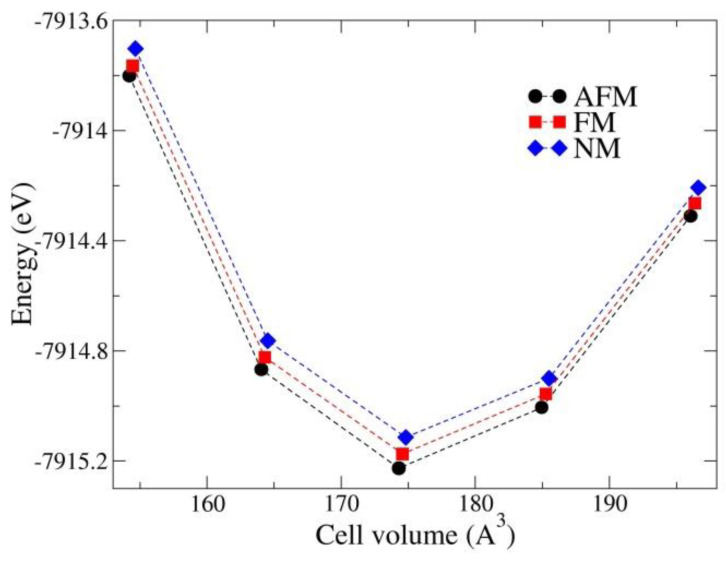
Variation in the total energy as a function of the cell volume calculated for the AFM, FM, and NM states of the CuPPD structure with the PBE method.

At first, the PBE method was used to calculate the relative stability of nonmagnetic (NM), antiferromagnetic, and ferromagnetic states. From the total energy analysis as a function of the volume cell (Figure 2), we found the AFM state energetically more favorable than the FM and NM states by about 51.57 and 111.65 meV, respectively. Therefore, all subsequent calculations are performed for the most stable AFM state.

The structural parameters of the relaxed CuPPD unit cell are presented in Table 1. One can observe that lattice parameters significantly depend on the calculations’ details. Interestingly, implementing the Hubbard terms *U* to the Cu d- and O p-orbitals in the optimization process caused some characteristic changes to the lattice parameters. The calculated lattice constants *b* and *c* decrease with the value of *U*, whereas a reverse trend is observed for the *a* lattice parameter. It, at first, decreases as *U* increases, and when *U* reaches a value close to 4 eV, the calculated *a* parameter increases. Moreover, the unit cell volume decreases within the PBE+U as a function of U. Unfortunately, it should be emphasized that there are no data available in the literature for possible comparison.

The calculated average bond lengths between copper (Cu), phosphorus (P), oxygen (O), and hydrogen (H) atoms of the Cu_2_P_2_O_5*_H_2_O structure are listed in Table 2. As can be observed, the equilibrium bond length of CuPPD becomes essentially smaller by applying the PBE+U method, from ~0.04% (U = 2 eV) to 2.54% (U = 8 eV), in comparison with the PBE potential.

**Figure 3:**
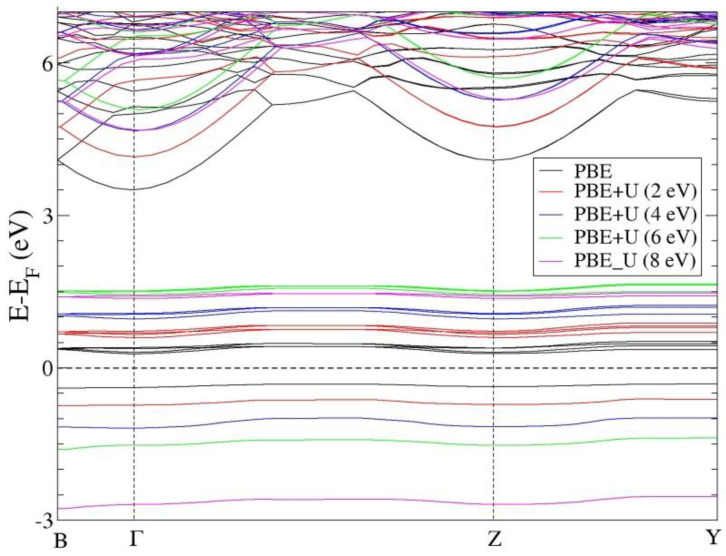
The electronic band structure of copper pyrophosphate calculated using PBE and PBE+U. The Fermi level (dashed line) was set to zero.

The electronic and magnetic properties are among the most studied features in novel materials, especially in areas linked to nanocrystalline QDs and nanostructured technologies. Consequently, the energy band structure, energy gap, or details of CuPPD’s partial density of states (PDOS) are of considerable interest. The electronic band structure of a CuPPD monoclinic structure computed within the spin-polarized approaches using the PBE and PBE+U methods is shown in Figure 3.

The results of PBE band calculations indicate the semiconductor-like character of CuPPD with two indirect band gaps of 0.59 and 3.92. The highest valence band of CuPPD is dominated by the transition metal Cu-3d and O-2p states, which show their strong hybridization. Furthermore, the electronic band structure analysis exhibits significant discrepancies above the Fermi energy, suggesting a strong correlation among electrons in Cu and O ions, leading to the necessity of using the Hubbard approximation in such systems. For this reason, we compared the effect of the U parameter value (0 < U < 8 eV) within PBE+U on the calculated electronic and magnetic properties of monoclinic CuPPD material. As shown in Figure 3 and Table 3, the PBE+U calculation improves the band gap values to a greater extent, overcoming the hybridization between the oxygen 2p and the transition metal copper 3d orbital. Furthermore, it is found that the calculated energy band gaps increase by raising the U parameter, and when the *U* reaches a value close to 8 eV, the second band gap value declines.

**Figure 4:**
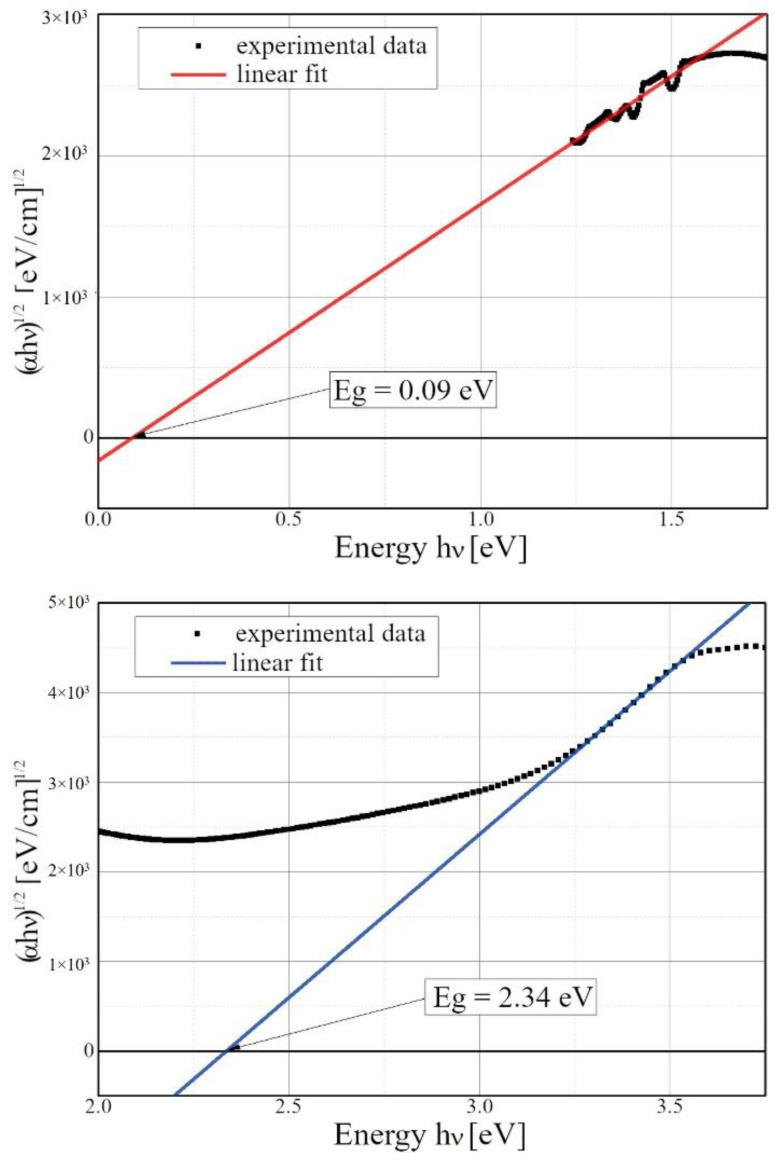
UV–VIS spectrum of monoclinic CuPPD and silver oxide nanocrystals restricted in SBA-15 mesoporous silica (black dots). According to Equation (2), linear fit indicates the energy gap width E_g_. The vertical axis is a square root of a product of absorption coefficient and the energy of radiation (n = 2).

The ultraviolet and visible (UV–VIS) absorption spectroscopy results were fitted, assuming that the copper pyrophosphate is an indirect band gap material (Figure 4). Thus, n = 2 was used for Equation (2). The fitting showed two band gaps with 0.09 eV and 2.34 eV. The first value is relatively small, and we believe it cannot be attributed to the copper pyrophosphate. Most probably it originates from other components of the mixture. Due to the sample preparation procedure, other ingredients (such as silver oxide) could not be removed entirely, thus giving rise to the UV–VIS absorption. On the other hand, the latter value of 2.34 eV fits our DFT/PBE+U calculations nicely. It is consistent with the DFT-calculated first band for U equal to 4.64 eV (values were obtained from linear interpolation of U from 2 to 8 eV). However, the results of our studies are based on a comparison of DFT values computed at 0 K and experimental data received at room temperature, and it is not evident whether temperature-induced changes might influence comparison.

**Figure 5:**
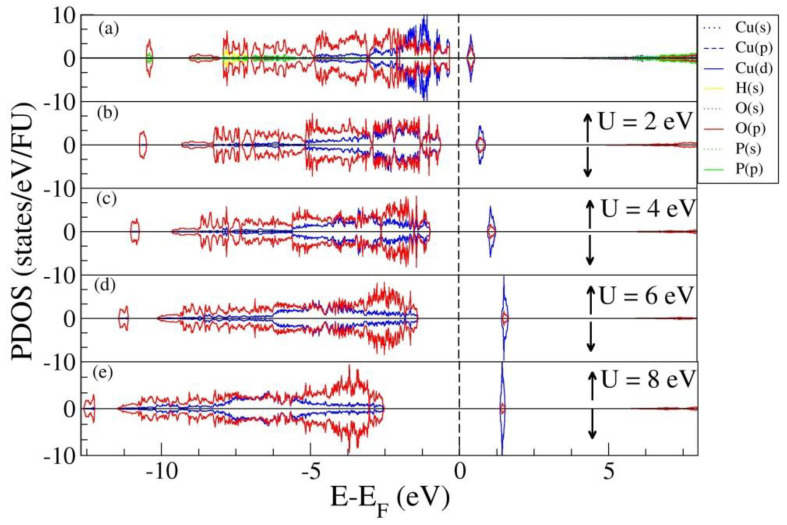
Partial density of states per formula unit (FU) of copper pyrophosphate using PBE and PBE+U methods in the AFM phase. The vertical dot line shows the position of the Fermi level.

Analysis of projected density of states (PDOS) (Figure 5) was performed to determine the electronic band’s modification due to the addition of Hubbard terms. PBE calculations in Figure 5a reveal that the electronic states near the top of the valence band are mainly filled by Cu-d and O-p electrons. Therefore, for clarity, Figure 5b–e shows only mentioned states. Closer inspection of PDOS spectra obtained by PBE and PBE+U methods also shows an additional peak above the Fermi level in both spin channels, and this feature is formed by the Cu-d and O-p states. Furthermore, PBE+U calculation results indicate that the application of U shifted the electronic states of CuPPD and consequently extended both energy gaps. 

In addition, to address the effect of the U correction on the description of the CuPPD compound’s magnetic properties, we compare PBE and PBE+U methods and obtain magnetic moment for Cu and O elements, the total magnetic moment per cell, listed in Table 3. The calculated total magnetic moment of copper pyrophosphate compound equals 6.91 μB for the PBE method.

Our results indicate that the total magnetic moments generally originate from the Cu ions with a small contribution of O ions. The PBE+U functional showed significant changes in the magnetic properties of the compound. The magnetic moment on Cu ion changed from 5.45 μB to 6.85 μB with the value of *U*, whereas a different trend was observed for the O ion, where magnetic moments decreased from 0.48 μB to 0.20 μB. It was observed that the PBE+U method predicts a larger value for the magnetic moment because Hubbard formalism reduces the PBE delocalization error between Cu-3d and O-2p states.

## 4. Conclusions

To summarize, we used the DFT calculations within PBE and PBE+U schemes to characterize the atomic structure and predict the electric and magnetic properties of monoclinic CuPPD compounds restricted in SBA-15 mesoporous silica. We found the AFM phase to be the most stable state compared with the FM and NM phases. Applying the Hubbard correction for the Cu and O atoms provides essential changes in the magnetic and electronic properties of CuPPD crystal. We found that variation of the magnetization of our material increases with the value of U and mainly comes from Cu’s partially filled d orbitals. The magnetic moments change from 6.91 μB (PBE) to 8.07 μB (PBE+U, U = 8 eV). Furthermore, the DFT-calculated indirect band gap energy at Γ point of the first Brillouin zone changes from 0.59 eV and 3.92 eV (PBE) to 3.89 eV and 4.04 eV (PBE+U, U = 8 eV). We found that PBE+U (U = 4.64 eV) obtained energy band gaps in good agreement with an experimental optical energy band gap of 2.34 eV received by UV–VIS measurement. Interestingly, electronic structures indicated the presence of a narrow peak in the spin-up and spin-down channels concentrated around the Fermi energy originated from the Cu-3d and O-2p bands, suggesting that monoclinic CuPPD crystal exhibits semiconducting-like properties. We conclude that our PBE+U method is appropriate to calculate the structural, electronic, and magnetic properties of monoclinic CuPPD compounds. We hope that the result of our calculations contains information important to modern semiconductor technology.

## Figures and Tables

**Table 1:** Optimized lattice parameters and unit cell volume of monoclinic CuPPD estimated with PBE and PBE+U for AFM configuration.

Structural Parameters	PBE	PBE+U			
		U = 2 eV	U = 4 eV	U = 6 eV	U = 8 eV
a (Å)	11.466	11.435	11.439	11.452	11.472
b (Å)	10.204	10.032	9.924	9.762	9.689
c (Å)	6.123	6.115	6.111	6.105	6.113
V (Å^3^)formula unit	196.054	170.605	168.790	166.107	165.118

**Table 2:** Selected average bond lengths (Å) of copper pyrophosphate dihydrate material calculated with PBE and PBE+U.

Bonds	Interatomic Distances (A)				
	PBE	U = 2 eV	U = 4 eV	U = 6 eV	U = 8 eV
Cu_1_-P_1_	3.287	3.277	3.269	3.261	3.255
Cu_1_-O_2_	2.025	2.012	2.005	1.999	2.001
P_1_-O_2_	1.577	1.563	1.553	1.543	1.535
O_8_-H_1_	0.982	0.975	0.969	0.963	0.959

**Table 3:** Hubbard U parameter values for Cu (d) and O (p) orbitals, energy gap value, and magnetic moments, calculated by the DFT/PBE+U methodology in a spin-polarized case.

PBE+U	μ_Cu_	μ_O_	μ_tot_ (Bohr mag/Cell)	E_g_ (eV)
U = 2 eV	5.45	0.48	7.65	1.18, 4.6
U = 4 eV	5.97	0.39	7.88	1.92, 4.93
U = 6 eV	6.46	0.29	8.00	2.79, 5.29
U = 8 eV	6.85	0.20	8.07	3.89, 4.04

## Data Availability

Derived data supporting the findings of this study are available from the corresponding author.
